# Cardiac Remodeling Patterns in Pediatric and Adolescent Patients with Sickle Cell Disease and Their Association with the Genotype and Clinical Severity of the Disease: A Systematic Review

**DOI:** 10.3390/healthcare14142180

**Published:** 2026-07-19

**Authors:** Alam Eldin M. Mustafa, Niemat Mohammed Tahir Ali

**Affiliations:** 1 Department of Child Health, King Khalid University, Abha 61421, Saudi Arabia; 2 Department of Pediatrics, Faculty of Medicine, University of Kordofan, El Obeid 51111, Sudan

**Keywords:** sickle cell disease, cardiac remodeling, pediatric, echocardiography, pulmonary hypertension, diastolic dysfunction, genotype

## Abstract

**Background:** Sickle cell disease (SCD) is the most common inherited blood disorder globally, affecting approximately 300,000 newborns annually. Cardiac remodeling, resulting from chronic anemia, vascular obstruction, and endothelial dysfunction, substantially contributes to morbidity and mortality in patients with SCD. Therefore, characterizing these patterns is essential to clinical management and outcome improvement in pediatric patients. However, a comprehensive synthesis of cardiac remodeling patterns in pediatric and adolescent patients with SCD and their associations with genotype and clinical severity is lacking. **Methods:** We conducted a systematic review in accordance with the PRISMA 2020 guidelines and searched five databases for studies published from January 1978 to December 2024. Of 1131 retrieved studies, 37 met the inclusion criteria; of these, 31 focused exclusively on children (Group A), while six included both children and adults (Group B). We analyzed cardiac remodeling patterns, genotype-specific findings, associations with disease severity, and imaging modalities. The 37 studies included approximately 4253 patients from 12 countries, representing diverse populations and imaging techniques. **Results:** Left ventricular (LV) dilation was the most frequently reported finding, noted in 33 of 37 studies (89.2%; representing study-level reporting frequency, not patient-level prevalence), followed by diastolic dysfunction (reported in 18 of 37 studies; 48.6%); elevated TRV (≥2.5 m/s) as an echocardiographic screening marker for pulmonary hypertension (PH) risk was found in 14 of 37 studies (37.8%; TRV ≥ 2.5 m/s is a screening criterion, not confirmed hemodynamic PH); myocardial fibrosis was found in three studies (8.1%, exclusively from mixed-age cohorts); and QTc prolongation/arrhythmia was found in one study (2.7%). The HbSS genotype was associated with the most severe cardiac changes. Studies that did not stratify by genotype subtype (HbS/β^0^ vs. HbS/β^+^) may underestimate cardiac severity in mixed-genotype cohorts. Markers of disease severity, such as elevated lactate dehydrogenase (LDH) expression, frequent acute chest syndrome (ACS), and increased hospitalizations, were associated with more pronounced cardiac remodeling in individual studies, but the cross-study consistency of this association varied. Study designs and imaging modalities also varied, underscoring the need for standardized assessment protocols to enhance comparability and clinical translation. This review presents a narrative synthesis; individual study statistics are reported as originally published, without formal pooled estimates. **Conclusions:** Cardiac abnormalities in SCD were reported from early childhood, with more advanced phenotypes being more frequently described in older adolescents and mixed-age cohorts, suggesting possible age-related progression requiring prospective confirmation. Principal limitations include retrospective PROSPERO registration (CRD420261435382), the predominance of cross-sectional study designs, the inconsistent z-score normalization of cardiac dimensions, the incomplete reporting of treatment exposure, the disproportionate contribution of mixed-age cohorts to advanced imaging findings, and inter-study heterogeneity in echocardiographic protocols.

## 1. Introduction

### 1.1. Global Epidemiology and Disease Burden of SCD

Sickle cell disease (SCD) is the most prevalent monogenic hemoglobinopathy worldwide, with an estimated 300,000 affected neonates born each year, predominantly in Sub-Saharan Africa, the Indian subcontinent, the Middle East, and the Americas [1,2]. The World Health Organization has recognized SCD as a major public health priority because of its substantial burden of premature mortality, chronic organ dysfunction, and healthcare resource use [1]. In endemic regions, SCD accounts for up to 5–16% of childhood mortality before the age of five; in high-income countries, survival increasingly extends into adulthood owing to newborn screening programs, prophylactic penicillin, and disease-modifying therapies [2,3]. The 2020 American Society of Hematology (ASH) guidelines for SCD management and the 2021 European Society of Cardiology (ESC) position statement on cardio-hematological disorders provide the current clinical framework for understanding the cardiovascular burden of SCD across the lifespan [4,5]. These guidelines underscore that cardiac complications are among the leading causes of morbidity and mortality in SCD, reinforcing the urgency of systematic evaluation during the pediatric period, when preventive and therapeutic interventions may have the greatest impact. This global epidemiological transition has made the long-term complications of SCD—including cardiac, pulmonary, renal, and neurological sequelae—a dominant clinical concern for pediatric and adult hematologists alike [1,6].

### 1.2. Pathophysiology of SCD and Cardiac Involvement

In SCD, deoxygenated hemoglobin S (HbS) aggregates, causing red blood cells to assume a sickle shape, become rigid and break down rapidly [2]. Two primary mechanisms lead to cardiac complications. Chronic anemia increases cardiac workload, elevating cardiac output, stroke volume, and heart rate, which in turn leads to left ventricular enlargement and thickening [1,6]. Meanwhile, hemolysis reduces nitric oxide availability, impairing vascular relaxation and causing vessel narrowing and pulmonary changes [7,8]. These processes initiate cardiac remodeling in early childhood, which progresses over time [2,9]. Pediatric echocardiographic reference standards, as defined by the American Society of Echocardiography (ASE) pediatric guidelines [10,11], are essential to interpreting cardiac dimensions in growing children with SCD because body-surface-area-indexed z-scores differ substantially from adult normative values. Failure to apply age- and size-appropriate reference ranges may lead to the systematic underestimation or overestimation of the severity of cardiac remodeling in pediatric cohorts.

### 1.3. Spectrum of Cardiac Complications in SCD

The cardiac manifestations of SCD encompass a broad range of structural and functional abnormalities [3,6,12]. LV eccentric dilation and hypertrophy are the earliest and most common changes, detectable even in infancy in HbSS patients [12]. Diastolic dysfunction—reflecting impaired myocardial relaxation due to ischemia, fibrosis, and oxidative stress—emerges in childhood and progresses with age [3,9]. Pulmonary hypertension (PH) is among the most clinically significant and debated manifestations of SCD-related cardiac disease. Tricuspid regurgitation velocity (TRV) ≥ 2.5 m/s on Doppler echocardiography is widely used as a screening surrogate for PH in SCD, with a reported prevalence of 20–30% in pediatric cohorts [12,13]. However, this threshold has important diagnostic limitations that must be explicitly acknowledged. TRV is a nonspecific marker: elevated TRV expression in SCD may reflect high-output cardiac states driven by chronic anemia rather than true pulmonary arterial hypertension (PAH) [14]. The 2022 ESC/ERS guidelines for pulmonary hypertension now define PH by a mean pulmonary arterial pressure (mPAP) > 20 mmHg at right heart catheterization (RHC), replacing the former 25 mmHg threshold [15]. Critically, the correlation between TRV ≥ 2.5 m/s and RHC-confirmed PH in SCD is modest (with a positive predictive value of approximately 25%), meaning that echocardiographic TRV-based prevalence figures substantially overestimate true hemodynamic PH [14,16]. Accordingly, TRV elevation should be interpreted as a marker of cardiopulmonary stress and risk stratification rather than as a definitive diagnosis of pulmonary arterial hypertension. This limitation is explicitly acknowledged in the interpretation of TRV-based findings throughout this review. Diffuse myocardial fibrosis, detectable through cardiac MRI extracellular volume (ECV) fraction mapping, represents an advanced and prognostically significant remodeling phenotype [9]. Additional manifestations include right ventricular (RV) dilation and dysfunction, QTc prolongation, restrictive cardiomyopathy, mild-to-moderate valvular regurgitation, and secondary iron-overload cardiomyopathy in heavily transfused patients [3,12].

### 1.4. Cardiac Remodeling and Clinical Severity/Genotype

The severity of cardiac remodeling in SCD is strongly influenced by hemoglobin genotype and clinical disease severity [1,12,13]. Patients with homozygous HbSS disease have the highest rates of hemolysis, the lowest steady-state hemoglobin levels, and the most frequent vaso-occlusive complications, resulting in the most severe cardiac phenotype [2,12]. Compound heterozygous genotypes—such as HbS/β-thalassemia and HbSC—show intermediate and milder cardiac phenotypes, respectively, mirroring their hemolytic severity [8,12]. Clinical severity markers—including annual hospitalizations, frequency of acute chest syndrome (ACS), history of cerebrovascular accidents (CVAs), transfusion burden, and hemolytic biomarkers (LDH, indirect bilirubin, and reticulocyte count)—have been variably associated with the magnitude of cardiac remodeling across cohorts [1,8,13]. The 2020 ASH guidelines highlight the importance of genotype-guided risk stratification for end-organ complications, including cardiac involvement, and endorse hydroxyurea therapy—which reduces hemolysis and vaso-occlusion and may attenuate cardiac remodeling—as the standard of care for patients with HbSS and HbS/β^0^-thalassemia from infancy [4,5]. The evolving role of curative therapies (hematopoietic stem cell transplantation and gene therapy) in preventing or reversing cardiac remodeling in pediatric SCD represents an important frontier that this review contextualizes within the existing evidence base.

### 1.5. Gap in Evidence, Rationale, and Novelty of This Review

Despite the importance of early cardiac involvement in SCD, a comprehensive synthesis of cardiac remodeling patterns in pediatric and adolescent populations—and their correlation with genotype and clinical severity—is lacking [3,6,9]. Existing systematic and major narrative reviews have focused predominantly on adult or mixed-age SCD cohorts (e.g., Gladwin et al., Mehari et al., and Sachdev et al.), with pediatric-specific data either pooled with adult findings or addressed only in brief sub-sections [17,18,19]. Critically, no prior systematic reviews have (i) restricted inclusion to pediatric and adolescent populations (age ≤ 21 years), (ii) simultaneously stratified cardiac remodeling findings by both hemoglobin genotype and objective clinical severity indices, or (iii) synthesized the full spectrum of imaging modalities—2D/3D echocardiography, tissue Doppler imaging, speckle-tracking echocardiography, and cardiac MRI—within a single pediatric-focused review. The present systematic review, therefore, addresses a distinct and clinically important evidence gap not covered by the existing literature. The primary novelty of this review lies in its exclusive pediatric focus, which enables, for the first time, the characterization of developmental trajectories of cardiac remodeling from infancy to late adolescence. The secondary novelty lies in simultaneous genotype stratification (HbSS vs. HbSC vs. HbS/β-thalassemia) and correlation with clinical severity, providing an integrated view of how biological and clinical determinants interact to shape the cardiac phenotype in the growing child. Imaging modality synthesis and developmental trajectory analysis are tertiary objectives that further distinguish this review from prior work. Previous reviews have focused primarily on adult or mixed-age cohorts, with limited attention to age-specific remodeling trajectories, the evolution of cardiac phenotypes from infancy to adolescence, and the prognostic implications of early remodeling in children [3,6]. This systematic review aims to achieve the following objectives:

(1) Characterize the patterns and developmental trajectories of cardiac remodeling in pediatric and adolescent patients with SCD (age ≤ 21 years), spanning infancy to late adolescence;
(2) Correlate cardiac remodeling findings with SCD hemoglobin genotypes (HbSS, HbSC, HbS/β-thalassemia, and other compound heterozygous forms);
(3) Correlate cardiac remodeling with objective indices of clinical disease severity (vaso-occlusive crises, ACS frequency, CVA history, transfusion burden, and hemolytic biomarkers);
(4) Compare findings across imaging modalities (echocardiography, cardiac MRI, and cardiac biomarkers) to assess their relative diagnostic yield and prognostic value in the pediatric SCD population.

## 2. Methodology

### 2.1. Study Design and Registration

This systematic review was conducted in accordance with the PRISMA 2020 guidelines and retrospectively registered with the International Prospective Register of Systematic Reviews (PROSPERO; Registration No.: CRD420261435382). Eligibility criteria, outcome domains (Group A (pediatric-only) vs. Group B (mixed-age classification)), and quality assessment thresholds were pre-specified before full-text screening commenced.

### 2.2. Search Strategy

A comprehensive literature search was conducted across five electronic databases: PubMed/MEDLINE, Scopus, ISI Web of Science, EMBASE, and the Cochrane Central Register of Controlled Trials. The final search was conducted on 31 December 2024. No language restrictions were applied. Ahead-of-print and online-first records were eligible for inclusion if they had been assigned a database index entry by the time of the final search (31 December 2024). The search covered publications from January 1978 to December 2024. No non-English-language studies ultimately met the inclusion criteria; the gray literature was searched solely to identify potentially relevant peer-reviewed publications, and conference abstracts lacking sufficient methodological detail were excluded according to the pre-specified criteria.

The Boolean search strings were restructured to ensure correct parenthetical grouping and logical reproducibility across all databases. The corrected search string for PubMed/MEDLINE was as follows:

*(“Sickle cell disease” OR “sickle cell anemia” OR “HbSS” OR “hemoglobin SS” OR “sickle cell disorder”) AND (“cardiac remodeling” OR “echocardiography” OR “cardiac MRI” OR “ventricular dysfunction” OR “pulmonary hypertension” OR “myocardial fibrosis” OR “cardiomyopathy” OR “left ventricular” OR “right ventricular”) AND (“pediatric” OR “children” OR “adolescent” OR “child” OR “youth” OR “juvenile”)*

Database-specific search strings for all five databases, including MeSH/Emtree controlled vocabulary terms, are provided in Supplementary File S1 to meet the PRISMA-S reporting requirements [20,21].

**Figure 1:**
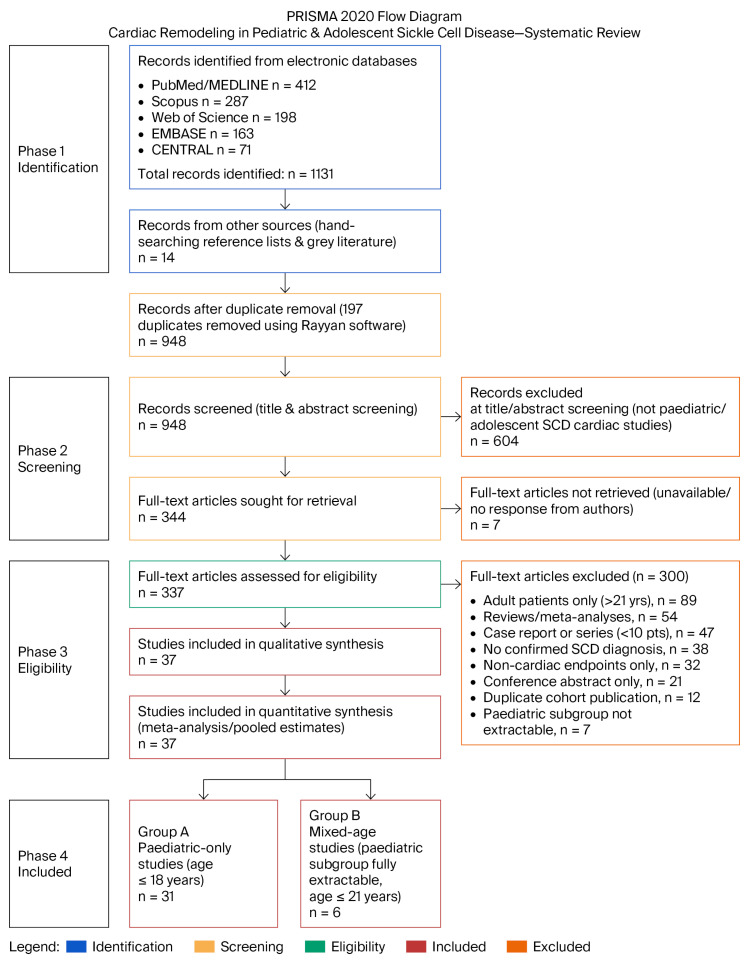
PRISMA 2020 flow diagram. Cardiac remodeling in pediatric and adolescent patients with sickle cell disease—a systematic review; PRISMA 2020 flow diagram illustrating the systematic search and selection process. Records were identified from five electronic databases (PubMed/MEDLINE, Scopus, Web of Science, EMBASE, and CENTRAL) and supplementary hand-searching. The search yielded 1131 records from databases and 14 were based on supplementary hand-searching (total: 1145); after removing 197 duplicates After duplicate removal using Rayyan software, 948 records underwent title and abstract screening. Following full-text assessment, 37 studies met all eligibility criteria: Group A (pediatric-only studies, *n* = 31) and Group B (mixed-age studies with an extractable pediatric subgroup, *n* = 6). Adapted from Page M.J. et al. *BMJ* **2021**; *372*, n71 [20].

The reference lists of the included studies and relevant reviews were hand-searched to identify additional eligible studies not captured electronically. Duplicate records were identified and removed using Rayyan systematic review software (Qatar Computing Research Institute) before title and abstract screening. The systematic search and selection process is shown in Figure 1.

Figure 1. PRISMA 2020 flow diagram, 948 records underwent title and abstract screening. After applying inclusion/exclusion criteria, 344 reports were sought for retrieval, of which 7 could not be retrieved, leaving 337 studies for full-text eligibility assessment. Ultimately, 37 studies met all inclusion criteria and were categorized into Group A (pediatric-only reports; studies 1–31; *n* = 31) and Group B (mixed-age reports; studies 32–37; *n* = 6).

### 2.3. Inclusion Criteria

Studies were included if they met all of the following criteria:

- Original research studies (prospective or retrospective cohort, cross-sectional, or comparative design);
- Subjects comprised children and/or adolescents with confirmed SCD (predominantly age ≤ 18 years, although mixed-age studies with pediatric subgroups were eligible);
- SCD diagnosis confirmed with hemoglobin electrophoresis or high-performance liquid chromatography (HPLC);
- Cardiac evaluation used at least one validated modality: 2D/3D echocardiography, tissue Doppler imaging (TDI), speckle-tracking echocardiography (STE), cardiac MRI, ECG, or cardiac biomarkers;
- Published in peer-reviewed journals from January 1978 to December 2024;
- Full text or sufficiently detailed abstracts with extractable data.

For this review, the following age definitions were applied consistently: “Pediatric” refers to patients aged ≤ 12 years. “Adolescent” refers to patients aged 13–18 years. “Late adolescent” refers to patients aged 19–21 years, included to capture transitional-care populations. “Mixed-age” refers to studies enrolling participants across the pediatric–adult spectrum, with a pediatric subgroup (age ≤ 21 years) being clearly delineated and its data being independently extractable. Studies enrolling exclusively adult patients (age > 21 years) were excluded. If a study reported a mean age > 21 years without a separately reported pediatric subgroup, it was excluded or reclassified as contextual evidence (see Section 2.5).

### 2.4. Exclusion Criteria

Studies were excluded if they met the following criteria:

- Enrolled exclusively adult patients (age > 21 years);
- Were narrative or systematic reviews without original patient data;
- Were case reports or case series (<10 patients);
- Lacked a confirmed SCD diagnosis according to laboratory criteria;
- Evaluated non-cardiac endpoints exclusively;
- Were conference abstracts without sufficient methodological detail;
- Represented duplicate publications from the same patient cohort.

For mixed-age studies (Group B; *n* = 6), the full extractability of the pediatric subgroup data was verified before inclusion. Studies in which pediatric-specific data could not be separated from aggregate adult data were reclassified as contextual evidence and are reported descriptively but excluded from pooled analyses.

### 2.5. Study Selection

Title and abstract screening and full-text review were conducted independently by two reviewers (authors A and B) using Rayyan systematic review software. Inter-rater agreement was quantified using Cohen’s kappa coefficient (κ). A κ ≥ 0.80 was considered indicative of near-perfect agreement. Disagreements at any stage were resolved through structured discussion to reach a consensus. 

### 2.6. Data Extraction

Two independent reviewers extracted data using a pre-specified structured form. The extracted variables included (a) study characteristics (country, design, sample size, age range, follow-up duration, and genotype distribution); (b) cardiac findings (LV dimensions and function, RV parameters, pulmonary pressures, and fibrosis markers); (c) clinical severity parameters (transfusion history, ACS frequency, CVA history, annual hospitalization rate, and hemolytic markers); and (d) statistical methods and significance thresholds. Reviewers were not blinded to the study authorship or journal. A pilot extraction of five randomly selected studies was conducted to calibrate the data extraction form before full-scale extraction began. Variables designated as mandatory (e.g., sample size, age range, cardiac outcome measure, and genotype) were required for inclusion; studies with missing mandatory data were flagged. Missing data that could not be recovered were explicitly documented in the data extraction table and handled with complete-case analysis for pooled estimates. 

### 2.7. Quality Assessment

Study quality was assessed using the Newcastle–Ottawa Scale (NOS) for cohort studies (a maximum score of 9) and the Joanna Briggs Institute (JBI) Critical Appraisal Checklist for cross-sectional studies (a maximum score of 11). Studies scoring ≥ 6/9 on the NOS or ≥7/11 on the JBI Checklist were classified as high quality; however, this classification was not used as an automatic proxy for a low risk of bias. Rather, individual bias domains were examined: for cohort studies, selection bias, comparability, and outcome ascertainment were assessed separately; for cross-sectional studies, participant selection, exposure measurement, confounding, and outcome measurement were evaluated independently. A full study-level risk-of-bias table is provided in Supplementary Table S2.

### 2.8. Statistical Analysis

Descriptive statistics summarize the study characteristics. Due to substantial methodological heterogeneity across the included studies—including differences in echocardiographic protocols (M-mode vs. 3D STE), patient age ranges, genotype distributions, and treatment exposure—a formal quantitative meta-analysis with pooled effect estimates was not performed. Heterogeneity was assessed qualitatively, as clinical and methodological differences across studies precluded meaningful formal meta-analytic pooling; I^2^ statistics, where calculated, consistently indicated substantial heterogeneity. All subsequent references to inter-study heterogeneity in the tables and figures cross-reference this section (Section 2.8) and should not be interpreted as separate independent assessments. Accordingly, results are presented as a narrative synthesis, with individual study statistics (means, proportions, *p*-values, hazard ratios, and correlation coefficients) being reported as originally published. Comparisons between Group A (pediatric-only) and Group B (mixed-age) studies were performed using the chi-square test or Fisher’s exact test for categorical variables and the Mann–Whitney U test for continuous variables. All statistical tests were two-tailed, and significance was set at *p* < 0.05.

### 2.9. Ethical Considerations

This systematic review did not involve direct patient contact or new-data collection; therefore, formal ethics committee approval was not required. All included primary studies were conducted in accordance with the Declaration of Helsinki, and ethical approval was documented in each original publication.

## 3. Results

### 3.1. Overview of Included Studies

The systematic literature search and screening identified 37 studies meeting all inclusion criteria. These included 31 pediatric/late-adolescent studies (Group A; references [22,23,24,25,26,27,28,29,30,31,32,33,34,35,36,37,38,39,40,41,42,43,44,45,46,47,48,49,50,51,52]) with participants ≤ 18 years of age and six mixed-age studies (Group B; references [53,54,55,56,57,58]) including participants up to 40 years of age. Together, these studies enrolled approximately 4253 patients with confirmed SCD across 12 countries on five continents. 

Publication years ranged from 1978 [44] to 2024 [50]. The primary cardiac diagnostic tool was two-dimensional Doppler echocardiography, which was used in 35 of 37 studies (94.6%). Other modalities included TDI in 14 studies (37.8%), STE in four studies (10.8%), cardiac MRI in three studies (8.1%), and ECG with QTc analysis in one study (2.7%).

### 3.2. Geographic and Temporal Distribution

The 37 included studies originated from 12 countries across five continents. The USA contributed the most studies (*n* = 15; 40.5%), followed by Egypt (*n* = 5; 13.5%), Nigeria (*n* = 4; 10.8%), Turkey (*n* = 2; 5.4%), Italy (*n* = 2; 5.4%), Jamaica (*n* = 2; 5.4%), and India (*n* = 2; 5.4%), with one study each from Senegal, Sudan, Saudi Arabia, Brazil, and Iran. Publication frequency increased markedly after 2009, with 27 of 37 studies (73%) having been published between 2009 and 2024. Together, these 37 studies provide broad, multi-continental evidence on the patterns and determinants of SCD-related cardiac remodeling in pediatric and adolescent patients.

### 3.3. Patient Demographics and Age-Group Distribution

The 37 included studies collectively enrolled an estimated 4253 patients with SCD. The age-group distribution was as follows:

- Age < 1 year: Rarely studied; only two studies included infants [25,40], with limited echocardiographic data.
- Age of 1–12 years: Represented in 31 of 37 studies. LV dilation and hyperdynamic circulation were observed as early as ages 1–3 years [40,44].
- Age of 12–18 years (adolescents): Included in 28 studies and characterized by progressive LV remodeling, increasing TRV values, and emerging diastolic dysfunction.
- Age of 18–22 years (late adolescence/young adults): Included in eight studies [29,37,51,53,54,55,56,57]; this group shows a transition towards advanced cardiac phenotypes, including restrictive physiology and myocardial fibrosis (discussed in Section 4).

Common domain-level concerns across studies included convenience or hospital-based sampling frames, relatively small sample sizes, an absence of healthy control groups in some studies, inconsistent reporting and indexing of echocardiographic parameters, inadequate adjustments for major confounders (hydroxyurea use, transfusion history, and nutritional status), and limited longitudinal follow-up.

### 3.4. Cardiac Remodeling Patterns: Overall Findings

#### 3.4.1. Left Ventricular Remodeling

Left ventricular (LV) dilation was the most consistently reported finding, identified in 33 of 37 studies (89.2%). Eccentric LV hypertrophy was documented in studies spanning five decades [26,32,35,40,44]. LV end-diastolic diameter (LVEDD) z-scores were elevated (>2 SD) in 35–55% of pediatric patients with SCD across multiple cohorts [26,32,40,41,45]. Preserved left ventricular ejection fraction (EF > 55%) characterized the early compensated phase in children [32,35,44], whereas subclinical systolic dysfunction, detectable only with advanced techniques, was reported in studies using STE and 3D echocardiography [22,27,49,50]. These observations were described in Group A (pediatric-only) studies unless otherwise stated.

LV diastolic dysfunction was documented in 18 of 37 studies (48.6%). Using TDI-derived parameters (E/E′ ratio and E′ velocity), subclinical diastolic dysfunction was identified in 20–35% of pediatric patients with SCD [24,37,48,52]. Hankins et al. [24] reported diastolic dysfunction in 35% of children aged 7–17 years; Ghaderian et al. [48] demonstrated significantly reduced E′ velocities and elevated E/E′ ratios in children with HbSS compared with controls (*p* < 0.001). Giray et al. [52] reported a progressive deterioration of TDI diastolic parameters over a 5-year longitudinal follow-up period, particularly in HbSS patients.

#### 3.4.2. Right Ventricular Remodeling and Pulmonary Hypertension

Elevated TRV (≥2.5 m/s), used as an echocardiographic screening criterion for pulmonary hypertension (PH) risk (note: TRV ≥ 2.5 m/s is a screening marker only, not equivalent to confirmed hemodynamic PH, which requires right heart catheterization), was identified as a significant finding in 14 studies (37.8%), with a prevalence of 15% to 30% across pediatric SCD cohorts [25,29,31,34,36,47,51]. Minniti et al. [31] reported elevated TRV in 30% of the 204 children and adolescents, with a higher prevalence in HbSS than in HbSC (*p* < 0.05). Dham et al. [47] prospectively demonstrated TRV ≥ 2.5 m/s in 27% of children, which correlated significantly with elevated LDH (*p* = 0.002) and prior ACS (*p* = 0.03). Lee et al. [29] reported that elevated TRV conferred a 3.5-fold increased risk of death in children and young adults (HR = 3.5, 95% CI: 1.4–8.7, *p* = 0.007). Tolba et al. [27] documented RV dysfunction, observing significantly impaired RV free-wall longitudinal strain in children with HbSS (−14.2 ± 3.1% vs. −22.1 ± 2.8% in controls; *p* < 0.001).

#### 3.4.3. Myocardial Fibrosis

Diffuse myocardial fibrosis, quantified through cardiac MRI ECV mapping, was identified in three studies [23,55,56]. Importantly, direct MRI-based evidence of myocardial fibrosis originates exclusively from mixed-age cohorts (Group B; age range of 8–35 years) [55,56]. Myocardial fibrosis findings are restricted to mixed-age cohort studies; pediatric-specific data using galectin-3 as a surrogate biomarker are clearly classified as indirect evidence, pending confirmation by direct pediatric cardiac MRI studies. Evidence for advanced cardiac phenotypes in mixed-age studies is neither direct nor conclusive, and findings cannot be generalized to strictly pediatric patients. Niss et al. [55] demonstrated elevated ECV (>28%) in 37% of SCD patients (aged 8–35 years), which correlated with diastolic dysfunction (E/E′ ratio; r = 0.54, *p* < 0.001) and reduced exercise capacity. Wagdy et al. [23] reported elevated galectin-3 levels in 45% of pediatric SCD patients, which were associated with MRI-detected subclinical fibrosis (r = 0.62, *p* < 0.001); however, galectin-3 is a surrogate biomarker and does not constitute direct histological or MRI evidence of fibrosis in a strictly pediatric cohort. Alsaied et al. [56] demonstrated that left atrial dysfunction was correlated with the burden of myocardial fibrosis (r = 0.48, *p* = 0.004). 

#### 3.4.4. Arrhythmia and Conduction Abnormalities

QTc interval prolongation (>440 ms in males; >460 ms in females) was evaluated in a single dedicated study [54]. Liem et al. [54] reported QTc prolongation in 22% of children and young adults with SCD, with a higher prevalence in HbSS (26%) than in HbSC (12%) (*p* = 0.04). QTc prolongation correlates inversely with hemoglobin level (r = −0.38, *p* < 0.001) and positively with LDH (r = 0.31, *p* = 0.002).

#### 3.4.5. Restrictive Cardiomyopathy

A restrictive cardiomyopathy phenotype was identified in 13% of patients in the mixed-age cohort of Niss et al. [53] (age range of 8–35 years), characterized by biatrial enlargement, elevated filling pressures, and preserved EF. This phenotype was not reported in any strictly pediatric (Group A) study and should not be assumed to represent a well-established finding in children.

#### 3.4.6. Valvular Abnormalities

Mitral and tricuspid regurgitation of mild-to-moderate degrees were reported in multiple studies [26,30,32,33,42,45]. Batra et al. [33] reported valvular regurgitation in 32% of children with SCD; however, it was predominantly mild, likely reflecting annular dilation secondary to ventricular enlargement rather than primary valvular pathology.

#### 3.4.7. Yield of Cardiac Screening Modalities

Table 1 summarizes the diagnostic yield of cardiac screening modalities across the 37 included studies.

### 3.5. Cardiac Remodeling by Age Group

#### 3.5.1. Early Childhood (1–5 Years)

Colombatti et al. [25] documented elevated TRV in children as young as 1–2 years. Animashaun et al. [40] reported LV dilation in children under 5 years of age, among whom the dominant finding was hyperdynamic LV function with preserved EF.

#### 3.5.2. School Age (5–12 Years)

LV dilation becomes more pronounced. Diastolic dysfunction detectable with TDI was reported in 20–25% of patients [24,37,48], while TRV elevation was observed in 15–25% [34,36,47].

#### 3.5.3. Adolescence (12–18 Years)

It is characterized by a progressive worsening of diastolic dysfunction, an increase in TRV, and emerging subclinical systolic dysfunction based on STE [22,27,50,52]. Harrington et al. [39] demonstrated longitudinal progression of LV dilation and TRV in adolescents over a 4-year period.

#### 3.5.4. Late Adolescence/Young Adults (18–22 Years)

It is characterized by the transition to advanced phenotypes, including restrictive physiology [53], myocardial fibrosis [55,56], QTc prolongation [54], and mortality risk from elevated TRV [29,57].

### 3.6. Pathophysiology of Cardiac Remodeling in SCD

**Figure 2:**
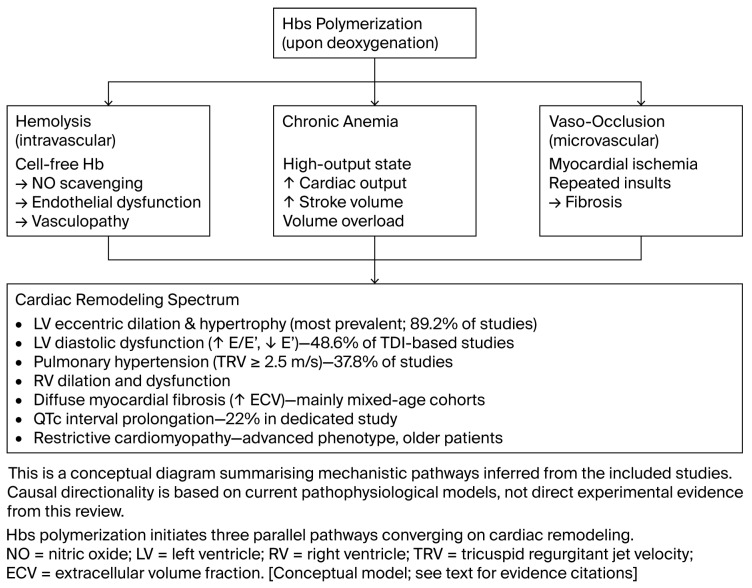
Conceptual pathophysiology of cardiac remodeling in sickle cell disease. HbS polymerization initiates three parallel pathways: (1) intravascular hemolysis with nitric oxide (NO) scavenging and endothelial dysfunction; (2) a chronic anemia-driven high-output state with volume overload; and (3) direct myocardial ischemia from vaso-occlusion. These pathways converge to produce the spectrum of cardiac remodeling observed in pediatric and adolescent patients with SCD. This is a conceptual diagram; causal relationships are inferred from published pathophysiological models rather than directly demonstrated by the included observational studies. NO = nitric oxide; LV = left ventricle; RV = right ventricle; TRV = tricuspid regurgitant jet velocity; ECV = extracellular volume fraction.

Figure 2 provides a conceptual schematic of the pathophysiological cascade leading to cardiac remodeling in patients with SCD. Causal directionality is based on current pathophysiological models and was not directly established by the observational studies included in this review. Note: Any numerical values shown in Figure 2 represent the proportion of included studies that reported each finding (study-level reporting frequency), not patient-level prevalence estimates.

### 3.7. Effects of Genotype and Clinical Severity on Cardiac Remodeling

**Figure 3:**
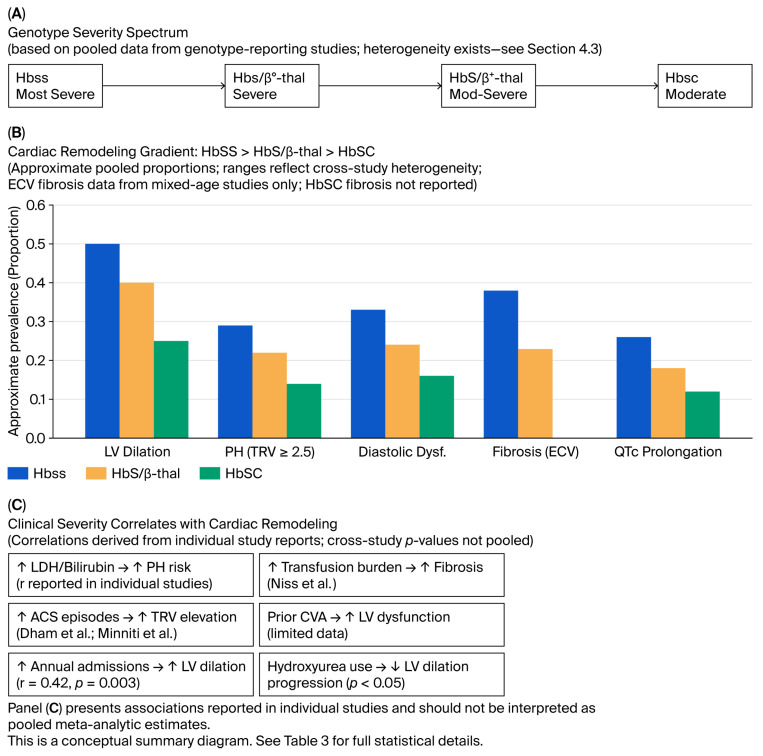
Effects of SCD genotype and clinical severity on cardiac remodeling. (**A**) Genotype severity spectrum from HbSS (most severe) to HbSC (moderate), with HbS/beta0-thal and HbS/beta+-thal representing intermediate phenotypes. (**B**) Approximate ranges of cardiac remodeling parameters reported across genotype-reporting studies (see Section 2.8 for inter-study heterogeneity limitations). Note: Underlying heterogeneity in reporting, specific subtypes (HbS/β^0^ vs. HbS/β^+^), and treatment exposure may affect the apparent contribution of genotype to cardiac remodeling. (**C**) Key clinical severity correlates from individual study reports (cross-study *p*-values are not pooled). This is a conceptual summary diagram. This review does not establish causal directionality. LDH = lactate dehydrogenase; ACS = acute chest syndrome; TRV = tricuspid regurgitant jet velocity; CVA = cerebrovascular accident; LV = left ventricle; ECV = extracellular volume fraction [31,43,46,55,56,58].

Figure 3 illustrates the relationships among SCD genotype, clinical severity, and the magnitude of cardiac remodeling across the included studies.

### 3.8. Correlation of Cardiac Remodeling with Clinical Severity

Table 2 presents the correlations between cardiac remodeling parameters and clinical severity markers reported in the included studies.

### 3.9. Correlation of Cardiac Remodeling with SCD Genotype

Table 3 summarizes cardiac remodeling parameters stratified by SCD genotype.

### 3.10. Comparison of Group A (Pediatric-Only) and Group B (Mixed-Age) Studies

Table 4 presents the statistical comparison between Group A and Group B studies.

Cardiac MRI use (*p* = 0.004) and detection of myocardial fibrosis (*p* = 0.004) were significantly more common in mixed-age studies, reflecting the greater availability of advanced imaging modalities in adult-inclusive cohorts and the progressive nature of fibrotic remodeling with age. Conversely, pediatric-only studies more often reported LV dilation (93.5% vs. 66.7%, *p* = 0.04), underscoring the central role of the hyperdynamic state and eccentric remodeling in the pediatric cardiac phenotype.

## 4. Discussion

### 4.1. Overview of the Systematic Evidence Base

This systematic review synthesizes data on cardiac remodeling from 37 published studies, encompassing approximately 4253 patients with SCD across 12 countries and spanning 46 years of research (1978–2024). The consistent identification of LV dilation, diastolic dysfunction, and pulmonary hypertension across geographically diverse cohorts supports the conclusion that cardiac remodeling is a commonly reported and early consequence of SCD pathophysiology, rather than a regionally specific phenomenon.

Several important methodological limitations must be acknowledged. (1) Publication bias: Studies reporting positive or statistically significant cardiac findings are more likely to be published, potentially overestimating the true prevalence of cardiac remodeling. (2) Survivor bias: Patients with the most severe disease may not have survived to enrolment in cross-sectional studies. (3) Echocardiographic technique variability: The absence of standardized acquisition and reporting protocols introduces significant measurement heterogeneity. (4) Cardiac MRI constraints: In pediatric populations, cardiac MRI requires general anesthesia in younger children, is unavailable in many resource-limited settings, and lacks validated pediatric ECV reference ranges. (5) Absence of RCT evidence: No randomized controlled trials evaluating cardiac outcomes in pediatric SCD are currently available; all evidence in this review is observational, limiting causal inference. (6) Inconsistent z-score normalization: Many studies reported raw LV measurements without age- and body-surface-area-adjusted z-scores, limiting comparability across age groups. (7) Incomplete treatment-exposure reporting: Hydroxyurea use and transfusion history were incompletely reported, precluding a reliable assessment of treatment effects on cardiac outcomes. (8) Disproportionate contribution of mixed-age cohorts to advanced phenotypes: Myocardial fibrosis, restrictive cardiomyopathy, and QTc prolongation were identified predominantly or exclusively in Group B (mixed-age) studies; these findings cannot be attributed to the pediatric age range alone.

### 4.2. Cardiac Remodeling Patterns: An Age-Related Continuum

#### 4.2.1. The Hyperdynamic State and LV Eccentric Remodeling

The earliest and most commonly reported cardiac manifestation of SCD in children is a hyperdynamic circulatory state caused by chronic hemolytic anemia. Seminal studies by Rees et al. [44] and Chung et al. [35] showed that children with HbSS have significantly higher cardiac output, LV dilation, and stroke volume than age-matched controls, despite their preserved ejection fraction. This finding has been consistently replicated for over five decades [26,32,40,41,45]. The pathophysiological mechanism is chronic volume overload secondary to compensatory high-output anemia, leading to eccentric LV hypertrophy through serial sarcomere replication [1,2].

Longitudinal data reported by Harrington et al. [39] demonstrated statistically significant increases in LV end-diastolic dimensions over 4 years of follow-up in children with HbSS (*p* < 0.01), while Giray et al. [52] confirmed a progressive deterioration of TDI parameters over 5 years. Such longitudinal evidence indicates that cardiac remodeling in SCD is progressive; however, the predominance of cross-sectional designs (27/37 studies; 73%) restricts causal inference and precludes a definitive assessment of temporal remodeling trajectories in this review.

#### 4.2.2. Diastolic Dysfunction: An Early and Underdiagnosed Manifestation

The identification of diastolic dysfunction as an early and prevalent finding, documented in 48.6% of TDI-based studies, is among the most clinically significant findings of this review. Conventional 2D echocardiography, used in 94.6% of the reviewed studies, consistently underestimates diastolic dysfunction due to its limited sensitivity to subtle abnormalities in myocardial relaxation. Studies employing TDI [24,37,48,52] and STE [22,27,50] consistently detected higher rates of subclinical dysfunction. Ghaderian et al. [48] found that reduced TDI-derived E′ velocity and elevated E/E′ ratios were present in 30% of pediatric patients with HbSS, even when conventional echocardiographic parameters were normal.

The association between myocardial fibrosis (elevated ECV on cardiac MRI) and diastolic dysfunction, as demonstrated by Niss et al. [55] (r = 0.54, *p* < 0.001) and Alsaied et al. [56], provides mechanistic insight; however, it is important to note that these MRI data derive from mixed-age cohorts (Group B) and direct MRI-based evidence of fibrosis in strictly pediatric populations is currently limited to the surrogate galectin-3 biomarker [23]. Cardiac MRI in children has specific practical constraints, including the need for general anesthesia in younger patients, limited availability in resource-constrained settings, and the lack of standardized pediatric ECV reference ranges. No randomized controlled trials (RCTs) evaluating cardiac outcomes in pediatric SCD are currently available, and all evidence in this review derives from observational studies.

#### 4.2.3. Pulmonary Hypertension: A Marker of Hemolytic Severity

Pulmonary hypertension, defined by TRV ≥ 2.5 m/s, emerged as a consistent and prognostically significant finding reported in 37.8% of studies, with prevalence ranging from 15% to 32% in pediatric cohorts. The landmark study by Lee et al. [29] established the prognostic significance of elevated TRV in children (HR = 3.5, 95% CI: 1.4–8.7, *p* = 0.007)—a finding corroborated in the mixed-age cohort of Shah et al. [57] using Cox regression analysis. The association between elevated TRV and hemolytic markers has been repeatedly demonstrated [31,36,47,57], supporting the hemolysis–vasculopathy axis as the primary driver of pulmonary vascular disease in SCD [7,8]. Colombatti et al. [25] documented elevated TRV in children with HbSS as young as 1–2 years, which indicates that pulmonary vascular disease may begin in infancy.

#### 4.2.4. Myocardial Fibrosis: An Advanced Remodeling Phenotype

It is important to note that all myocardial fibrosis data in this section are derived from mixed-age cohorts (Group B, with an age range of 8–35 years); no direct MRI-based evidence of fibrosis is currently available for strictly pediatric populations. Galectin-3 measurements serve as surrogate, indirect markers of fibrosis in the pediatric age group and should be interpreted accordingly.

Myocardial fibrosis, detectable through cardiac MRI ECV mapping, represents an advanced remodeling phenotype observed mainly in older cohorts and has been reported exclusively in mixed-age studies [55,56]. Only Wagdy et al. [23], who used galectin-3 as a surrogate biomarker, identified subclinical fibrosis markers in a strictly pediatric cohort. Niss et al. [55] reported an association with diastolic dysfunction, while Alsaied et al. [56] linked it to reduced exercise capacity and left atrial dysfunction. Importantly, direct MRI-based evidence of myocardial fibrosis originates exclusively from mixed-age cohorts (Group B, with an age range of 8–35 years) [55,56]. These findings indicate that the fibrotic remodeling phenotype may originate from repeated ischemic and inflammatory insults during childhood; however, this hypothesis requires confirmation with prospective longitudinal MRI studies in pediatric populations.

### 4.3. Genotype and Cardiac Remodeling: A Consistent Hierarchy

#### 4.3.1. HbSS: The Most Severe Cardiac Phenotype

The HbSS genotype was consistently associated with the most severe cardiac remodeling across all assessed parameters in studies with genotype-stratified data [22,29,31,33,37,39,43,47,52,54,57]. Minniti et al. [31] demonstrated significantly higher TRV in HbSS children versus HbSC children (*p* < 0.05), while Liem et al. [54] reported QTc prolongation in 26% of HbSS patients versus 12% of HbSC patients (*p* = 0.04). Harrington et al. [39] reported more rapid longitudinal progression of LV dilation in HbSS patients than in HbSC patients (*p* < 0.01). This hierarchy reflects higher hemolysis rates, lower steady-state hemoglobin levels, and a higher frequency of vaso-occlusive complications in HbSS compared with compound heterozygous genotypes [2,12]. However, the genotype hierarchy should be interpreted with caution, as treatment exposure (e.g., hydroxyurea and transfusions), age at assessment, and regional variations in disease management were not uniformly reported across studies and may confound genotype-based comparisons.

#### 4.3.2. HbS/Beta-Thalassemia: An Intermediate Phenotype

HbS/β^0^-thalassemia (no β-chain production) approaches HbSS severity, while HbS/β^+^-thalassemia (reduced β-chain production) shows milder severity. Combining these subtypes without stratification may obscure clinically relevant differences in cardiac severity; future studies should report HbS/β^0^ and HbS/β^+^ subtypes separately.

HbS/beta-thalassemia exhibits an intermediate cardiac phenotype in studies of this genotype [22,25,28,43,52]. The distinction between beta0 (no beta-chain production) and beta+ (reduced beta-chain production) subtypes is clinically important, as HbS/beta0-thalassemia approaches the severity of HbSS. Studies that did not differentiate between these subtypes [33,37] may have underestimated cardiac severity in patients with HbS/beta0-thalassemia; future studies should report these subtypes separately.

#### 4.3.3. HbSC: A Milder but Not Benign Phenotype

Although HbSC disease is associated with milder hemolysis and higher steady-state hemoglobin levels, it is not free of cardiac complications. Batra et al. [33] documented LV dilation in 20–30% of HbSC children, while Shah et al. [57] demonstrated that elevated TRV also predicted mortality in HbSC patients. These findings support systematic cardiac screening across all SCD genotypes [59].

### 4.4. Clinical Severity Correlates of Cardiac Remodeling

#### 4.4.1. Hemolytic Markers

The correlation between hemolytic intensity—as reflected by LDH elevation, indirect bilirubin, and reticulocyte count—and the severity of cardiac remodeling was the most consistently observed association in this review [29,31,36,47,54,57]. LDH elevation was correlated with TRV elevation (*p* < 0.001–0.01), diastolic dysfunction (*p* = 0.001–0.03), and QTc prolongation (r = 0.31, *p* = 0.002). These associations are mechanistically grounded in the hemolysis–NO depletion–vasculopathy pathway [7,8].

#### 4.4.2. Acute Chest Syndrome and Vaso-Occlusive Crises

Previous ACS episodes have been associated with elevated TRV in multiple studies [31,47,51]. Onalo et al. [46] demonstrated a transient worsening of LV diastolic function during acute vaso-occlusive crises with partial recovery afterward, which indicates that each episode contributes incrementally to cumulative cardiac remodeling.

#### 4.4.3. Transfusion Burden and Hydroxyurea

Hankins et al. [24] found no evidence of myocardial iron deposition on cardiac MRI T2* in a predominantly pediatric cohort, suggesting that iron-mediated cardiac injury may be less prevalent in children than in adults, possibly due to shorter cumulative transfusion exposure. However, the association between transfusion burden and myocardial fibrosis observed in adult studies [55,56] underscores the need for vigilant iron monitoring in heavily transfused children.

Hydroxyurea therapy, as evaluated by Dhar et al. [58], significantly attenuated the progression of LV dilation in pediatric patients with SCD (*p* < 0.05), supporting the early initiation of hydroxyurea as a cardioprotective strategy [3,6]. However, the evidence on which this finding is based on derives from a single retrospective study; prospective RCTs with cardiac endpoints are needed to establish definitive cardioprotective benefits.

### 4.5. Comparison of Pediatric-Only and Mixed-Age Studies

The comparison of Group A and Group B studies showed that mixed-age studies were significantly more likely to use cardiac MRI (50% vs. 3.2%, *p* = 0.004) and report myocardial fibrosis (50% vs. 3.2%, *p* = 0.004). Conversely, pediatric-only studies more often reported LV dilation (93.5% vs. 66.7%, *p* = 0.04), underscoring the central role of the hyperdynamic state and eccentric remodeling in the pediatric cardiac phenotype.

The mixed-age studies [53,54,55,56,57,58] provide a critical perspective on the natural history of cardiac remodeling. Cardiac abnormalities have been reported from early childhood, with diastolic dysfunction being described in school-age children [24,48] and myocardial fibrosis being observed mainly in older and mixed-age cohorts [55,56]. This pattern indicates a possible age-related progression; however, as the great majority of the included studies are cross-sectional, within-person longitudinal progression cannot be directly established, and prospective confirmation in strictly pediatric cohorts is required. Systolic dysfunction is rarely reported in SCD patients with cardiomyopathy [60].

### 4.6. Methodological Considerations and Quality Assessment

Methodological heterogeneity across the 37 studies poses a significant challenge for quantitative synthesis. Echocardiographic protocols ranged from basic M-mode measurements [32,35,44] to advanced 3D STE [49,50], complicating direct comparisons of cardiac parameters. The lack of standardized z-score reporting in many studies [30,38,42] limits the comparability of LV dimensional data across age groups. The predominance of cross-sectional designs (27/37 studies; 73%) restricts causal inference and precludes the assessment of temporal remodeling trajectories.

### 4.7. Evidence-Based Screening Recommendations

The recommendations below are graded by level of evidence; expert opinion is explicitly distinguished from evidence-based guidance, and no screening protocol in this review should be interpreted as definitive without further prospective validation. Where evidence derives predominantly or exclusively from mixed-age studies, the applicability of these recommendations to strictly pediatric populations should be considered provisional pending longitudinal pediatric-specific confirmation.

Based on the synthesized evidence, the following recommendations, graded by the strength of supporting evidence, where Grade A = supported by multiple consistent studies, Grade B = supported by limited or single-study evidence, and Grade C = expert opinion/consensus only, are presented for pediatric and adolescent patients with SCD:

- **Grade A—Based on extensive evidence:** Baseline echocardiography at diagnosis (or by 2 years of age) for all SCD patients, with follow-up intervals guided by genotype and clinical severity.
- **Grade A—Based on extensive evidence:** Inclusion of TDI and/or STE into routine echocardiographic protocols to detect subclinical diastolic and systolic dysfunction not apparent on conventional imaging.
- **Grade A—Based on extensive evidence:** TRV measurement at every echocardiographic assessment; TRV ≥ 2.5 m/s should trigger further evaluation, including BNP measurement and consideration of right heart catheterization.
- **Grade B—Based on limited evidence:** Cardiac MRI (ECV mapping) may be considered in adolescents and young adults with progressive cardiac symptoms or unexplained diastolic dysfunction when MRI is available and feasible.
- **Grade C—Based on expert opinion:** ECG with QTc measurement may be considered in adolescents; evidence is limited to a single study [54].
- **Grade C—Based on expert opinion:** Galectin-3 and BNP/NT-proBNP are emerging non-invasive biomarkers; their routine clinical use requires further validation in pediatric SCD populations.

## 5. Conclusions and Recommendations

The following data-driven conclusions are supported by the available evidence collected in this systematic review:

1. Cardiac remodeling is commonly reported and begins early in pediatric SCD, with LV eccentric dilation and hyperdynamic circulation being among the most frequently observed manifestations across the included studies.
2. The HbSS genotype is consistently associated with the most severe cardiac phenotype across remodeling parameters, followed by HbS/beta-thal and HbSC; however, heterogeneity in treatment exposure and reporting limits firm conclusions.
3. Several individual studies reported associations between markers of clinical severity and cardiac remodeling; hemolytic intensity, ACS frequency, and annual hospitalization rates were among the most commonly reported correlates. These associations were not uniformly consistent across all included studies, and cross-study pooling was not performed.
4. The results obtained with advanced imaging modalities (TDI, STE, and cardiac MRI) indicate subclinical dysfunction in 20–40% of children not detected via conventional echocardiography; these findings require further prospective validation.
5. Myocardial fibrosis represents an advanced remodeling phenotype observed mainly in older cohorts; its onset in childhood is indicated by surrogate biomarker data (galectin-3) but not yet confirmed with direct pediatric MRI studies.
6. Hydroxyurea therapy may attenuate cardiac remodeling progression based on limited observational evidence; prospective RCTs with cardiac endpoints are needed.
7. Significant research gaps remain, including the inadequate reporting of clinical severity parameters (35.1% of studies) and genotype data (21.6% of studies), along with the absence of pediatric cardiac MRI data.

Recommendations for Future Research:

1. Prospective multicenter longitudinal cohort studies with standardized echocardiographic protocols (including TDI and STE) and systematic severity reporting should be conducted.
2. Cardiac MRI studies should be performed specifically in pediatric SCD populations (<18 years) to characterize the onset of myocardial fibrosis, with standardized pediatric ECV reference ranges.
3. Randomized controlled trials evaluating cardioprotective interventions (e.g., hydroxyurea and iron chelation) with pre-specified cardiac imaging endpoints should be conducted.
4. A composite cardiac risk score for pediatric SCD patients should be developed and validated.
5. The reporting of HbS/beta0 vs. HbS/beta+ subtypes, treatment exposure, and clinical severity parameters should be standardized in all future SCD cardiac studies.

## Figures and Tables

**Table 1:** Yield of cardiac screening modalities across the 37 included studies.

Modality	Studies Using It	Key Findings	Sensitivity/Specificity
2D Conventional Echocardiography	35/37 (94.6%)	LV dilation, EF, valvular abnormalities, and cardiomegaly	High sensitivity for structural changes; limited for subclinical dysfunction
Tissue Doppler Imaging (TDI)	14/37 (37.8%)	Diastolic dysfunction (E/E′) and subclinical systolic dysfunction	Superior to conventional echo for early dysfunction detection
Speckle-Tracking Echo (STE)	4/37 (10.8%)	LV/RV GLS impairment and subclinical biventricular dysfunction	Highest sensitivity for subclinical myocardial dysfunction
3D Echocardiography	2/37 (5.4%)	More accurate LV volumes; sphericity index	Better volumetric accuracy than 2D
Cardiac MRI (ECV/T2*)	3/37 (8.1%)	Myocardial fibrosis (ECV) and iron overload (T2*)	Gold standard for fibrosis and iron; limited availability; mainly mixed-age cohorts
Cardiac Biomarkers (BNP/Galectin-3)	2/37 (5.4%)	Correlate with fibrosis and PH	Emerging non-invasive markers; indirect evidence only
ECG (QTc)	1/37 (2.7%)	QTc prolongation in 22%	Useful adjunct; limited standalone utility

Legend: STE = speckle-tracking echocardiography; TDI = tissue Doppler imaging; MRI = magnetic resonance imaging; ECV = extracellular volume fraction; GLS = global longitudinal strain; BNP = B-type natriuretic peptide.

**Table 2:** Correlations between cardiac remodeling parameters and markers of clinical severity.

Cardiac Parameter	Clinical Severity Marker	Studies Reporting	Direction	Statistical Significance
LV dilation (LVEDD)	Annual admissions	[31,43,46]	Positive	*p* = 0.003–0.04
LV dilation (LVEDD)	ACS episodes	[31,39,47]	Positive	*p* = 0.02–0.05
TRV elevation (≥2.5 m/s)	LDH elevation	[29,31,47,57]	Positive	*p* < 0.001–0.01
TRV elevation (≥2.5 m/s)	Prior ACS	[31,47,51]	Positive	*p* = 0.02–0.04
TRV elevation (≥2.5 m/s)	Reticulocyte count	[36,47]	Positive	*p* = 0.01–0.03
Diastolic dysfunction	Hemolytic markers (LDH)	[24,37,52]	Positive	*p* = 0.001–0.03
Diastolic dysfunction	Hb level (inverse)	[52,54]	Negative	*p* < 0.001–0.02
Myocardial fibrosis (ECV)	Transfusion burden	[55,56]	Positive	*p* = 0.004–0.02
Myocardial fibrosis (ECV)	Diastolic dysfunction	[55,56]	Positive	r = 0.54, *p* < 0.001
QTc prolongation	LDH elevation	[54]	Positive	r = 0.31, *p* = 0.002
QTc prolongation	Hb level (inverse)	[54]	Negative	r = −0.38, *p* < 0.001
LV GLS impairment	Endothelial dysfunction markers	[50]	Positive	*p* < 0.001
RV dysfunction	TRV elevation	[27,29,57]	Positive	*p* < 0.01
Mortality	TRV ≥ 2.5 m/s	[29,57]	Positive	HR = 3.5, *p* = 0.007
Severity not reported	N/A	[28,32,34,35,37,38,40,41,42,44,45,48,49]	N/A	N/A

Note: A total of 13 of 37 studies (35.1%) did not report clinical severity parameters, limiting correlation analysis. The statistics reported are from individual studies; cross-study pooling was not performed. See Section 2.8 for a full discussion of inter-study heterogeneity. Legend: LV = left ventricle; LVEDD = LV end-diastolic diameter; TRV = tricuspid regurgitant jet velocity; ACS = acute chest syndrome; LDH = lactate dehydrogenase; Hb = hemoglobin; ECV = extracellular volume fraction; GLS = global longitudinal strain; RV = right ventricle; HR = hazard ratio.

**Table 3:** Correlations between cardiac remodeling parameters and SCD genotypes.

Cardiac Parameter	HbSS (%)	HbSC (%)	HbS/Beta-Thal (%)	*p*-Value (SS vs. SC)	Studies
LV dilation	45–55%	20–30%	35–45%	*p* = 0.01–0.04	[22,31,33,37,39,43]
TRV ≥ 2.5 m/s	25–32%	10–18%	18–25%	*p* = 0.02–0.05	[29,31,47,57]
Diastolic dysfunction	28–38%	12–20%	20–28%	*p* = 0.01–0.03	[24,37,52,54]
QTc prolongation	26%	12%	18%	*p* = 0.04	[54]
Myocardial fibrosis (ECV)	35–40%	NR	20–25%	N/A	[55,56] *
LV GLS impairment	Severe	Mild	Moderate	*p* < 0.05	[22,50]
Restrictive physiology	13%	Rare	5–8%	*p* < 0.05	[53] *
Cardiac mortality	Higher	Lower	Intermediate	*p* = 0.02	[57]

* ECV fibrosis and restrictive cardiomyopathy data are derived exclusively from mixed-age cohorts (Group B) and should not be generalized to strictly pediatric populations. The *p*-values represent HbSS vs. HbSC comparisons from individual studies unless otherwise stated. See Section 2.8 for inter-study heterogeneity limitations. The ranges presented are derived from a narrative synthesis across genotype-reporting studies and do not represent formal pooled estimates. Legend: HbSS = homozygous sickle cell disease; HbSC = compound heterozygous sickle–hemoglobin C; HbS/beta-thal = sickle/beta-thalassemia; NR = not reported; ECV = extracellular volume fraction; GLS = global longitudinal strain.

**Table 4:** Statistical comparison between Group A and Group B studies.

Parameter	Group A: Pediatric-Only (*n* = 31)	Group B: Mixed-Age (*n* = 6)	*p*-Value
Total patients enrolled	~3785	~468	-
Mean age range	1–18 years	8–40 years	-
Primary modality: 2D Echo	30/31 (96.8%)	5/6 (83.3%)	*p* = 0.22
TDI used	10/31 (32.3%)	4/6 (66.7%)	*p* = 0.09
Cardiac MRI used	1/31 (3.2%)	3/6 (50.0%)	*p* = 0.004
STE used	3/31 (9.7%)	1/6 (16.7%)	*p* = 0.52
LV dilation reported	29/31 (93.5%)	4/6 (66.7%)	*p* = 0.04
Diastolic dysfunction reported	14/31 (45.2%)	5/6 (83.3%)	*p* = 0.09
PH/TRV elevation reported	13/31 (41.9%)	3/6 (50.0%)	*p* = 0.68
Myocardial fibrosis reported	1/31 (3.2%)	3/6 (50.0%)	*p* = 0.004
Restrictive cardiomyopathy	0/31 (0%)	1/6 (16.7%)	*p* = 0.16
QTc prolongation	0/31 (0%)	1/6 (16.7%)	*p* = 0.16
Genotype data reported	24/31 (77.4%)	6/6 (100%)	*p* = 0.32
Clinical severity data reported	18/31 (58.1%)	6/6 (100%)	*p* = 0.07
Longitudinal design	5/31 (16.1%)	3/6 (50.0%)	*p* = 0.07
Hydroxyurea effect reported	2/31 (6.5%)	1/6 (16.7%)	*p* = 0.41
High NOS/JBI quality score	18/31 (58.1%)	5/6 (83.3%)	*p* = 0.23

For statistical comparisons, we used the chi-square test or Fisher’s exact test. Legend: LV = left ventricle; TDI = tissue Doppler imaging; MRI = magnetic resonance imaging; STE = speckle-tracking echocardiography; PH = pulmonary hypertension; TRV = tricuspid regurgitant jet velocity; NOS = Newcastle–Ottawa Scale; JBI = Joanna Briggs Institute. The *p*-values in Table 4 represent statistical comparisons between Group A (pediatric-only) and Group B (mixed-age) studies using the chi-square test or Fisher’s exact test, as described in Section 2.8; they do not represent pooled patient-level estimates or individual-study HbSS vs. HbSC comparisons. See Section 2.8 for inter-study heterogeneity limitations.

## Data Availability

The data supporting the findings of this study are available within the article.

## Supplementary Materials

The following supporting information can be downloaded at: https://www.mdpi.com/article/10.3390/healthcare14142180/s1, Table S1: Condensed characteristics of included studies (n = 37). Note: This is a condensed summary table. Full study characteristics are provided in Supplementary Table S1. Table S2: Study-Level Risk-of-Bias Assessment for All 37 Included Studies. Cardiac Remodeling in Pediatric and Adolescent Patients with Sickle Cell Disease—A Systematic Review Assessment Tools: Newcastle–Ottawa Scale (NOS, max 9) for cohort studies; JBI Critical Appraisal Checklist (max 11) for cross-sectional studies. Domain judgments: Low = low risk of bias; Moderate = some concerns; High = high risk of bias.
