# Supplementary material for: Cardiac Remodeling Patterns in Pediatric and Adolescent Patients with Sickle Cell Disease and Their Association with the Genotype and Clinical Severity of the Disease: A Systematic Review
Source: Healthcare (Basel). 2026 Jul 19;14(14):2180. doi: 10.3390/healthcare14142180 (PMC13409839; doi:10.3390/healthcare14142180)
Supplement: Supplementary file 1 [file healthcare-14-02180-s001.zip › file S1.pdf]

Supplementary File S1

Database-Specific Boolean Search Strings for Systematic Literature Search

Systematic Review: The Pattern of Cardiac Remodeling in Pediatric and Adolescent Patients with Sickle Cell Disease

Search conducted: January 1978 – December 31, 2024 | Databases searched: 5 | No language restrictions applied

Legend and Abbreviations

This supplementary file presents the full database-specific Boolean search strings used in the systematic review, structured to comply with the PRISMA-S (Preferred Reporting Items for Systematic Reviews and Meta-Analyses — Literature Search extension) reporting guidelines. Each string is tailored to the syntax, controlled vocabulary, and field tags of its respective database.

Abbreviation / Tag	Meaning
`MeSH`	Medical Subject Headings (controlled vocabulary; PubMed/MEDLINE and Cochrane)
`Emtree`	Embase Tree (controlled vocabulary; EMBASE)
`[MeSH Terms]`	PubMed MeSH field tag — retrieves indexed MeSH headings and all narrower terms
`[Title/Abstract]`	PubMed free-text field tag — searches title and abstract fields
`/exp`	EMBASE Emtree explosion — includes the term and all narrower Emtree concepts
`:ti,ab,kw`	EMBASE/Cochrane — title, abstract, and keyword fields
`TITLE-ABS-KEY`	Scopus combined field tag — title, abstract, and author keywords
`TS=`	Web of Science Topic field — searches title, abstract, author keywords, Keywords Plus
`OR / AND`	Boolean operators (upper case required in all databases)
`[ ] / ' '`	Field tags: square brackets = PubMed; single quotes = EMBASE
`explode all trees`	Cochrane MeSH: retrieves the concept and all narrower hierarchical terms

1. PubMed/MEDLINE

Controlled vocabulary: Medical Subject Headings (MeSH). Field tags: [MeSH Terms] for indexed headings (all subheadings and narrower terms automatically retrieved); [Title/Abstract] for free-text terms. Boolean operators in UPPER CASE. Search interface: PubMed Advanced Search Builder. Date range filter applied: 1978/01/01–2024/12/31.

Concept Block 1 — Disease (Sickle Cell Disease)

```
("Anemia, Sickle Cell"[MeSH Terms]
   OR "sickle cell disease"[Title/Abstract]
   OR "sickle cell anemia"[Title/Abstract]
   OR "HbSS"[Title/Abstract]
   OR "hemoglobin SS"[Title/Abstract]
   OR "sickle cell disorder"[Title/Abstract])
```

Concept Block 2 — Cardiac Outcomes / Remodeling

```
("Cardiomegaly"[MeSH Terms]
   OR "Cardiomyopathies"[MeSH Terms]
   OR "Echocardiography"[MeSH Terms]
   OR "Magnetic Resonance Imaging"[MeSH Terms]
   OR "Hypertension, Pulmonary"[MeSH Terms]
   OR "Myocardial Fibrosis"[MeSH Terms]
   OR "Ventricular Dysfunction"[MeSH Terms]
   OR "Heart Ventricles"[MeSH Terms]
   OR "cardiac remodeling"[Title/Abstract]
   OR "echocardiography"[Title/Abstract]
   OR "cardiac MRI"[Title/Abstract]
   OR "ventricular dysfunction"[Title/Abstract]
   OR "pulmonary hypertension"[Title/Abstract]
   OR "myocardial fibrosis"[Title/Abstract]
   OR "cardiomyopathy"[Title/Abstract]
   OR "left ventricular"[Title/Abstract]
   OR "right ventricular"[Title/Abstract])
```

Concept Block 3 — Population (Pediatric / Adolescent)

```
("Child"[MeSH Terms]
   OR "Adolescent"[MeSH Terms]
   OR "Pediatrics"[MeSH Terms]
   OR "pediatric"[Title/Abstract]
   OR "children"[Title/Abstract]
   OR "adolescent"[Title/Abstract]
   OR "child"[Title/Abstract]
   OR "youth"[Title/Abstract]
   OR "juvenile"[Title/Abstract])
```

Final Combined PubMed/MEDLINE String

```
("Anemia, Sickle Cell"[MeSH Terms] OR "sickle cell disease"[Title/Abstract]
   OR "sickle cell anemia"[Title/Abstract] OR "HbSS"[Title/Abstract]
   OR "hemoglobin SS"[Title/Abstract] OR "sickle cell disorder"[Title/Abstract])
AND
("Cardiomegaly"[MeSH Terms] OR "Cardiomyopathies"[MeSH Terms]
   OR "Echocardiography"[MeSH Terms] OR "Magnetic Resonance Imaging"[MeSH Terms]
   OR "Hypertension, Pulmonary"[MeSH Terms] OR "Myocardial Fibrosis"[MeSH Terms]
   OR "Ventricular Dysfunction"[MeSH Terms] OR "Heart Ventricles"[MeSH Terms]
   OR "cardiac remodeling"[Title/Abstract] OR "echocardiography"[Title/Abstract]
   OR "cardiac MRI"[Title/Abstract] OR "ventricular dysfunction"[Title/Abstract]
   OR "pulmonary hypertension"[Title/Abstract] OR "myocardial
fibrosis"[Title/Abstract]
   OR "cardiomyopathy"[Title/Abstract] OR "left ventricular"[Title/Abstract]
   OR "right ventricular"[Title/Abstract])
AND
("Child"[MeSH Terms] OR "Adolescent"[MeSH Terms] OR "Pediatrics"[MeSH Terms]
   OR "pediatric"[Title/Abstract] OR "children"[Title/Abstract]
   OR "adolescent"[Title/Abstract] OR "child"[Title/Abstract]
   OR "youth"[Title/Abstract] OR "juvenile"[Title/Abstract])
```

---

2. EMBASE

Controlled vocabulary: Emtree (EMBASE's hierarchical thesaurus). Field notation: /exp = exploded Emtree term (includes all narrower concepts); :ti,ab,kw = free-text search in title, abstract, and author keywords. Single quotes enclose Emtree terms. Boolean operators in LOWER CASE. Search interface: Embase.com Advanced Search. Date range filter applied: 1978–2024.

Concept Block 1 — Disease (Sickle Cell Disease)

```
('sickle cell anemia'/exp
   OR 'sickle cell disease':ti,ab,kw
   OR 'HbSS':ti,ab,kw
   OR 'hemoglobin SS':ti,ab,kw
   OR 'sickle cell disorder':ti,ab,kw)
```

Concept Block 2 — Cardiac Outcomes / Remodeling

```
('heart remodeling'/exp
   OR 'echocardiography'/exp
   OR 'heart left ventricle'/exp
   OR 'heart right ventricle'/exp
   OR 'pulmonary hypertension'/exp
   OR 'myocardial fibrosis'/exp
   OR 'cardiomyopathy'/exp
   OR 'nuclear magnetic resonance imaging'/exp
   OR 'ventricular dysfunction':ti,ab,kw
   OR 'cardiac remodeling':ti,ab,kw
   OR 'cardiac MRI':ti,ab,kw
   OR 'left ventricular':ti,ab,kw
   OR 'right ventricular':ti,ab,kw)
```

Concept Block 3 — Population (Pediatric / Adolescent)

```
('child'/exp
   OR 'adolescent'/exp
   OR 'pediatrics'/exp
   OR 'pediatric':ti,ab,kw
   OR 'children':ti,ab,kw
   OR 'adolescent':ti,ab,kw
   OR 'youth':ti,ab,kw
   OR 'juvenile':ti,ab,kw)
```

Final Combined EMBASE String

```
('sickle cell anemia'/exp OR 'sickle cell disease':ti,ab,kw
   OR 'HbSS':ti,ab,kw OR 'hemoglobin SS':ti,ab,kw
   OR 'sickle cell disorder':ti,ab,kw)
AND
('heart remodeling'/exp OR 'echocardiography'/exp
   OR 'heart left ventricle'/exp OR 'heart right ventricle'/exp
   OR 'pulmonary hypertension'/exp OR 'myocardial fibrosis'/exp
   OR 'cardiomyopathy'/exp OR 'nuclear magnetic resonance imaging'/exp
   OR 'ventricular dysfunction':ti,ab,kw OR 'cardiac remodeling':ti,ab,kw
   OR 'cardiac MRI':ti,ab,kw OR 'left ventricular':ti,ab,kw
   OR 'right ventricular':ti,ab,kw)
AND
('child'/exp OR 'adolescent'/exp OR 'pediatrics'/exp
   OR 'pediatric':ti,ab,kw OR 'children':ti,ab,kw
   OR 'adolescent':ti,ab,kw OR 'youth':ti,ab,kw
   OR 'juvenile':ti,ab,kw)
```

---

3. Scopus

Scopus does not use an externally controlled vocabulary equivalent to MeSH. Free-text terms are searched in title, abstract, and author-defined keywords via the TITLE-ABS-KEY field tag. Search interface: Scopus Advanced Document Search. Date range: PUBYEAR > 1977 AND PUBYEAR < 2025. Document types: articles, reviews, conference papers, book chapters.

Final Combined Scopus String

```
TITLE-ABS-KEY(
   ("sickle cell disease" OR "sickle cell anemia" OR "HbSS"
      OR "hemoglobin SS" OR "sickle cell disorder")
   AND
   ("cardiac remodeling" OR "echocardiography" OR "cardiac MRI"
      OR "magnetic resonance imaging" OR "ventricular dysfunction"
      OR "pulmonary hypertension" OR "myocardial fibrosis"
      OR "cardiomyopathy" OR "left ventricular" OR "right ventricular")
```

```
   AND
   ("pediatric" OR "children" OR "adolescent" OR "child"
      OR "youth" OR "juvenile")
)
AND PUBYEAR > 1977 AND PUBYEAR < 2025
```

---

4. ISI Web of Science (Core Collection)

Web of Science Core Collection uses the Topic (TS=) field tag, which searches title, abstract, author keywords, and Keywords Plus simultaneously. No external controlled vocabulary is required; the Keywords Plus index partially compensates by adding terms from cited references. Boolean operators in UPPER CASE. Search interface: Web of Science Advanced Search. Publication Years filter: 1978–2024. Databases included: SCI-EXPANDED, SSCI, ESCI, CPCI-S.

Final Combined Web of Science String

```
TS=(
   ("sickle cell disease" OR "sickle cell anemia" OR "HbSS"
      OR "hemoglobin SS" OR "sickle cell disorder")
   AND
   ("cardiac remodeling" OR "echocardiography" OR "cardiac MRI"
      OR "ventricular dysfunction" OR "pulmonary hypertension"
      OR "myocardial fibrosis" OR "cardiomyopathy"
      OR "left ventricular" OR "right ventricular")
   AND
   ("pediatric" OR "children" OR "adolescent" OR "child"
      OR "youth" OR "juvenile")
)
AND PY=(1978-2024)
```

---

5. Cochrane Central Register of Controlled Trials (CENTRAL)

Controlled vocabulary: Medical Subject Headings (MeSH), shared with PubMed. The notation 'explode all trees' retrieves the specified MeSH heading and all narrower (child) terms within its hierarchy. Free-text field tags: :ti,ab,kw (title, abstract, and keyword). Search interface: Cochrane Library Advanced Search. Issue searched: Issue 12, December 2024.

Line-by-Line Search Strategy

```
#1   MeSH descriptor: [Anemia, Sickle Cell] explode all trees
#2   "sickle cell disease":ti,ab,kw
#3   "sickle cell anemia":ti,ab,kw
#4   "HbSS":ti,ab,kw
#5   "hemoglobin SS":ti,ab,kw
#6   "sickle cell disorder":ti,ab,kw
#7   #1 OR #2 OR #3 OR #4 OR #5 OR #6

#8   MeSH descriptor: [Cardiomegaly] explode all trees
#9   MeSH descriptor: [Cardiomyopathies] explode all trees
```

```
#10  MeSH descriptor: [Echocardiography] explode all trees
#11  MeSH descriptor: [Hypertension, Pulmonary] explode all trees
#12  MeSH descriptor: [Myocardial Fibrosis] explode all trees
#13  MeSH descriptor: [Ventricular Dysfunction] explode all trees
#14  MeSH descriptor: [Heart Ventricles] explode all trees
#15  "cardiac remodeling":ti,ab,kw
#16  "echocardiography":ti,ab,kw
#17  "cardiac MRI":ti,ab,kw
#18  "ventricular dysfunction":ti,ab,kw
#19  "pulmonary hypertension":ti,ab,kw
#20  "myocardial fibrosis":ti,ab,kw
#21  "cardiomyopathy":ti,ab,kw
#22  "left ventricular":ti,ab,kw
#23  "right ventricular":ti,ab,kw
#24  #8 OR #9 OR #10 OR #11 OR #12 OR #13 OR #14 OR #15
       OR #16 OR #17 OR #18 OR #19 OR #20 OR #21 OR #22 OR #23

#25  MeSH descriptor: [Child] explode all trees
#26  MeSH descriptor: [Adolescent] explode all trees
#27  MeSH descriptor: [Pediatrics] explode all trees
#28  "pediatric":ti,ab,kw
#29  "children":ti,ab,kw
#30  "adolescent":ti,ab,kw
#31  "child":ti,ab,kw
#32  "youth":ti,ab,kw
#33  "juvenile":ti,ab,kw
#34  #25 OR #26 OR #27 OR #28 OR #29 OR #30 OR #31 OR #32 OR #33

#35  #7 AND #24 AND #34
```

PRISMA-S Reporting Compliance

This supplementary file was prepared in accordance with the PRISMA-S (Preferred Reporting Items for Systematic Reviews and Meta-Analyses — Literature Search) extension checklist (Rethlefsen et al., 2021). The following PRISMA-S items are addressed by this file:

PRISMA-S Item	How Addressed in This File
Item 6	All databases, registers, and websites searched are named and described.
Item 7	The date of each search is reported (final search: December 31, 2024).
Item 8	Full search strategies for all databases are provided with no abbreviation.
Item 9	Controlled vocabulary (MeSH / Emtree) terms are reported for each database.
Item 10	Keywords and free-text terms are reported alongside controlled vocabulary.
Item 11	Boolean operators and field tags are explicitly stated for each string.
Item 12	Date range limits and language restrictions (none applied) are documented.

Reference: Rethlefsen ML, Kirtley S, Waffenschmidt S, et al. PRISMA-S: an extension to the PRISMA Statement for Reporting Literature Searches in Systematic Reviews. Systematic Reviews. 2021;10(1):39. https://doi.org/10.1186/s13643-020-01542-z
