# Supplementary material for: Cardiac Remodeling Patterns in Pediatric and Adolescent Patients with Sickle Cell Disease and Their Association with the Genotype and Clinical Severity of the Disease: A Systematic Review
Source: Healthcare (Basel). 2026 Jul 19;14(14):2180. doi: 10.3390/healthcare14142180 (PMC13409839; doi:10.3390/healthcare14142180)
Supplement: Supplementary file 1 [file healthcare-14-02180-s001.zip › tables.pdf]

Table S1. Condensed characteristics of included studies (*n* = 37). Note: This is a condensed summary table. Full study characteristics are provided in Supplementary Table S1.

SN [Ref]	First Author (Year)	Country	Design	N	Age Range	Genotype	Cardiac Modality	Key Finding	Severity
**Group A: Pediatric-Only Studies (*n* = 31)**									
1 [10]	Sabatini (2022)	Italy	Prosp. cross-sect.	40	2–18 yr	HbSS, HbSC, HbS/b-thal	2D Echo, TDI, and STE	LV dilation and systolic/diastolic dysfunction; RV strain abnormalities	Partial
2 [11]	Wagdy (2023)	Egypt	Cross-sect.	60	3–18 yr	HbSS predominantly	Echo + MRI + Galectin-3	Subclinical fibrosis by MRI; LV diastolic dysfunction; elevated galectin-3	Partial
3 [12]	Hankins (2010)	USA	Cross-sect.	76	7–17 yr	HbSS	Echo + MRI (T2*)	Diastolic dysfunction 35%; no myocardial iron deposition	Not reported
4 [13]	Colombatti (2010)	Italy	Prosp. cross-sect.	51	1–10 yr	HbSS, HbS/b-thal	Echo (TRV)	Elevated TRV in 25%; PH risk in young children	Partial
5 [14]	Chinawa (2021)	Nigeria	Cross-sect.	80	5–15 yr	HbSS	2D Echo and M-mode	LV dilation; increased LV mass; annular enlargement	Not reported
6 [15]	Tolba (2017)	Egypt	Cross-sect.	40	4–17 yr	HbSS	2D Echo + STE (RV)	RV free-wall strain impaired; TAPSE reduced	Partial
7 [16]	Arslankoylu (2010)	Turkey	Cross-sect.	30	4–16 yr	HbSS, HbS/b-thal	2D Echo + MPI	Elevated Tei index; subclinical biventricular dysfunction	Not reported
8 [17]	Lee (2009)	USA	Retro. cohort	147	2–20 yr	HbSS, HbSC, and HbS/b-thal	Echo (TRV)	TRV ≥ 2.5 m/s -> 3.5× increased mortality risk	Partial
9 [18]	Kane (2001)	Senegal	Cross-sect.	50	2–15 yr	HbSS	2D Echo	LV dilation; hyperdynamic state; MR in 20%	Not reported
10 [19]	Minniti (2009)	USA	Cross-sect.	204	3–20 yr	HbSS, HbSC, and HbS/b-thal	Echo (TRV)	Elevated TRV in 30%; higher in HbSS vs. HbSC ($p < 0.05$)	Partial
11 [20]	Lester (1990)	USA	Cross-sect.	50	6–18 yr	HbSS	2D Echo and M-mode	LV dilation and eccentric hypertrophy; hyperdynamic LV	Not reported
12 [21]	Batra (2002)	USA	Retro. cohort	67	1–18 yr	HbSS, HbSC, and HbS/b-thal	2D Echo and M-mode	LV dilation in 46%; RV dilation in 28%	Partial
13 [22]	Patel (2016)	India	Cross-sect.	60	2–18 yr	HbSS	Echo (TRV and RVSP)	PH prevalence 18.3%	Not reported
14 [23]	Chung (1987)	Jamaica	Cross-sect.	70	6–16 yr	HbSS	2D Echo and M-mode	Elevated cardiac output; LV dilation; normal systolic function	Not reported
15 [24]	Lamina (2019)	Nigeria	Cross-sect.	90	5–17 yr	HbSS	Echo (Doppler TRV)	Elevated mean PAP in 22%; TRV ≥ 2.5 m/s in 20%	Partial
16 [25]	Allen (2019)	USA	Prosp. cross-sect.	120	1–21 yr	HbSS, HbSC, and HbS/b-thal	2D Echo and TDI	Diastolic dysfunction 28%; LV dilation 35%	Partial
17 [26]	El Sayed (2017)	Egypt	Cross-sect.	50	3–16 yr	HbSS	2D Echo and M-mode	LV dilation; increased LVEDV	Not reported
18 [27]	Harrington (2017)	USA	Longit. prosp.	55	4–18 yr	HbSS and HbSC	2D Echo	Progressive LV dilation; TRV progression HbSS > HbSC	Partial
19 [28]	Animasahun (2010)	Nigeria	Cross-sect.	80	1–15 yr	HbSS	2D Echo and M-mode	LV dilation in 55%; elevated LVEDD z-score	Not reported
20 [29]	Cipolotti (2001)	Brazil	Cross-sect.	40	5–15 yr	HbSS	2D Echo and M-mode	LV dilation; increased LA diameter; preserved EF	Not reported
21 [30]	Ali (2012)	Sudan	Cross-sect.	50	2–14 yr	HbSS	2D Echo	LV dilation; pericardial effusion in 8%	Not reported
22 [31]	Waggass (2023)	Saudi Arabia	Retro. cohort	122	2–18 yr	HbSS, HbSC, and HbS/b-thal	2D Echo	Cardiomegaly 42%; LV dilation 38%; PH 15%	Partial
23 [32]	Rees (1978)	Jamaica	Cross-sect.	40	6–16 yr	HbSS	2D Echo and M-mode	Elevated cardiac output; LV dilation; hyperdynamic circulation	Not reported
24 [33]	Tidake (2015)	India	Cross-sect.	50	5–15 yr	HbSS	2D Echo and M-mode	LV dilation; preserved systolic function; mild MR	Not reported

25 [34]	Onalo (2020)	Nigeria	Prosp. comparative	60	5–16 yr	HbSS	2D Echo (crisis/post)	Worsening LV function during crisis; transient diastolic dysfunction	Partial
26 [35]	Dham (2009)	USA	Prosp. cohort	85	3–18 yr	HbSS, HbSC, and HbS/b-thal	Echo (TRV and RVSP)	TRV ≥ 2.5 m/s in 27%; correlates with LDH elevation	Partial
27 [36]	Ghaderian (2012)	Iran	Cross-sect.	40	5–15 yr	HbSS	2D Echo + TDI	Reduced E′; elevated E/E′; subclinical LV diastolic dysfunction	Not reported
28 [37]	Tolba (2015)	Egypt	Cross-sect.	45	4–16 yr	HbSS	3D Echo	Increased LV volumes; reduced EF vs. 2D; elevated sphericity index	Not reported
29 [38]	Abdelmassih (2024)	Egypt	Prosp. cross-sect.	35	6–18 yr	HbSS	3D STE + TDI	Endothelial dysfunction correlates with LV GLS impairment	Partial
30 [39]	Lilje (2017)	USA	Prosp. cross-sect.	75	2–21 yr	HbSS, HbSC, and HbS/b-thal	Echo (TRV) + BNP	BNP elevation predicts invasive PH confirmation	Partial
31 [40]	Giray (2023)	Turkey	Longit. prosp.	48	3–18 yr	HbSS and HbS/b-thal	TDI + Doppler	Progressive TDI deterioration over 5 years; earlier in HbSS	Partial
**Group B: Mixed-Age Studies (*n* = 6)**									
32 [41]	Niss (2016)	USA	Retro. cohort	55	8–35 yr	HbSS	Echo + cardiac MRI	Restrictive cardiomyopathy in 13%; associated with diastolic dysfunction and fibrosis	Partial
33 [42]	Liem (2009)	USA	Cross-sect.	102	10–30 yr	HbSS, HbSC, and HbS/b-thal	ECG (QTc)	Prolonged QTc in 22%; higher in HbSS	Partial
34 [43]	Niss (2017)	USA	Retro. cohort	60	8–35 yr	HbSS	Cardiac MRI (ECV)	Diffuse myocardial fibrosis (elevated ECV) in 37%	Partial
35 [44]	Alsaied (2020)	USA	Cross-sect.	50	10–35 yr	HbSS	Echo + cardiac MRI	LA dysfunction correlates with myocardial fibrosis	Partial
36 [45]	Shah (2021)	USA	Retro. cohort	143	10–40 yr	HbSS, HbSC, and HbS/b-thal	Echo (TRV) + TDI	TRV and TDI parameters predict mortality	Partial
37 [46]	Dhar (2021)	USA	Retro. longit.	58	4–21 yr	HbSS	2D Echo	Hydroxyurea reduces LV dilation progression	Partial

Legend: Group A = pediatric-only (studies 1–31); Group B = mixed-age (studies 32–37); HbSS = homozygous sickle cell; HbSC = compound heterozygous; HbS/b-thal = sickle/beta-thalassemia; TDI = tissue Doppler imaging; STE = speckle-tracking echocardiography; MPI = myocardial performance index; EF = ejection fraction; LV = left ventricle; RV = right ventricle; TRV = tricuspid regurgitant jet velocity; PH = pulmonary hypertension; NR = not reported. Full study data in Supplementary Table S1.

Table S2. Study-level risk-of-bias assessment for all 37 included studies. Cardiac remodeling in pediatric and adolescent patients with sickle cell disease—a systematic review assessment. Tools: Newcastle–Ottawa Scale (NOS, max 9) for cohort studies and JBI Critical Appraisal Checklist (max 11) for cross-sectional studies. Domain judgments: Low = low risk of bias; Moderate = some concerns; High = high risk of bias.

Study No.	First Author, Year	Country	Study Design	Appraisal Tool and Score	Selection Bias	Comparability/Confounding	Outcome/Measurement	Blinding/Attrition	Overall Risk of Bias
**GROUP A—Pediatric-Only Studies (*n* = 31)**									
1	Sabatini et al., 2022	Italy	Prospective cross-sectional	JBI 8/11	Low	Low	Low	Low	Low
2	Wagdy et al., 2023	Egypt	Cross-sectional	JBI 8/11	Low	Low	Low	Low	Low
3	Hankins et al., 2010	USA	Cross-sectional	JBI 9/11	Low	Low	Low	Low	Low
4	Colombatti et al., 2010	Italy	Prospective cross-sectional	JBI 9/11	Low	Low	Low	Low	Low
5	Chinawa et al., 2021	Nigeria	Cross-sectional	JBI 7/11	Low	Moderate	Low	Moderate	Moderate
6	Tolba et al., 2017	Egypt	Cross-sectional	JBI 8/11	Low	Low	Low	Low	Low
7	Arslankoylu et al., 2010	Turkey	Cross-sectional	JBI 8/11	Low	Low	Low	Low	Low

8	Lee et al., 2009	USA	Retrospective cohort	NOS 8/9	Low	Low	Low	Low	Low
9	Kane et al., 2001	Senegal	Cross-sectional	JBI 7/11	Low	Moderate	Low	Moderate	Moderate
10	Minniti et al., 2009	USA	Cross-sectional	JBI 9/11	Low	Low	Low	Low	Low
11	Lester et al., 1990	USA	Cross-sectional	JBI 7/11	Low	Low	Low	Moderate	Low
12	Batra et al., 2002	USA	Retrospective cohort	NOS 7/9	Low	Low	Low	Low	Low
13	Patel et al., 2016	India	Cross-sectional	JBI 8/11	Low	Low	Low	Low	Low
14	Chung et al., 1987	Jamaica	Cross-sectional	JBI 8/11	Low	Low	Low	Low	Low
15	Lamina et al., 2019	Nigeria	Cross-sectional	JBI 8/11	Low	Low	Low	Low	Low
16	Allen et al., 2019	USA	Prospective cross-sectional	JBI 9/11	Low	Low	Low	Low	Low
17	El-Sayed et al., 2017	Egypt	Cross-sectional	JBI 7/11	Low	Low	Low	Moderate	Moderate
18	Harrington et al., 2017	USA	Prospective longitudinal	NOS 8/9	Low	Low	Low	Low	Low
19	Animasahun et al., 2010	Nigeria	Cross-sectional	JBI 7/11	Low	Moderate	Low	Moderate	Moderate
20	Cipolotti et al., 2001	Brazil	Cross-sectional	JBI 8/11	Low	Low	Low	Low	Low
21	Ali et al., 2012	Sudan	Cross-sectional	JBI 7/11	Low	Moderate	Low	Moderate	Moderate
22	Waggass et al., 2023	Saudi Arabia	Retrospective cohort	NOS 7/9	Low	Low	Low	Low	Low
23	Rees et al., 1978	Jamaica	Cross-sectional	JBI 7/11	Low	Moderate	Low	Moderate	Moderate
24	Tidake et al., 2015	India	Cross-sectional	JBI 7/11	Low	Low	Low	Moderate	Low
25	Onalo et al., 2020	Nigeria	Prospective comparative	JBI 8/11	Low	Low	Low	Low	Low
26	Dham et al., 2009	USA	Prospective cohort	NOS 8/9	Low	Low	Low	Low	Low
27	Ghaderian et al., 2012	Iran	Cross-sectional	JBI 8/11	Low	Low	Low	Low	Low
28	Tolba et al., 2015	Egypt	Cross-sectional	JBI 8/11	Low	Low	Low	Low	Low
29	AbdelMassih et al., 2024	Egypt	Prospective cross-sectional	JBI 9/11	Low	Low	Low	Low	Low
30	Lilje et al., 2017	USA	Prospective cross-sectional	JBI 9/11	Low	Low	Low	Low	Low
31	Giray et al., 2023	Turkey	Prospective longitudinal	NOS 8/9	Low	Low	Low	Low	Low
**GROUP B—Mixed-Age Studies with Extractable Pediatric Subgroup (*n* = 6)**									
32	Niss et al., 2016	USA	Retrospective cohort	NOS 8/9	Low	Low	Low	Low	Low
33	Liem et al., 2009	USA	Cross-sectional	JBI 8/11	Low	Low	Low	Low	Low
34	Niss et al., 2017	USA	Retrospective cohort	NOS 8/9	Low	Low	Low	Low	Low
35	Alsaied et al., 2020	USA	Cross-sectional	JBI 9/11	Low	Low	Low	Low	Low
36	Shah et al., 2021	USA	Retrospective cohort	NOS 8/9	Low	Low	Low	Low	Low
37	Dhar et al., 2021	USA	Retrospective longitudinal	NOS 7/9	Low	Low	Low	Low	Low

Legend: NOS = Newcastle–Ottawa Scale; JBI = Joanna Briggs Institute. Selection bias: representativeness of cohort/sample, sampling method, and response rate. Comparability/confounding: control for age, sex, SCD genotype, hydroxyurea use, and transfusion history. Outcome/measurement: validity and reliability of cardiac assessment modality and blinding of outcome assessors. Blinding/attrition: blinding of personnel and completeness of follow-up/missing data handling. Overall risk of bias: Low = all key domains rated low; Moderate: ≥ 1 domain rated moderate, with none high; High: ≥ 1 domain rated high. Color coding: Green = low risk; Yellow = moderate risk; Red = high risk.
